# Elimination of oncogenic cells that regulate epithelial homeostasis in *Drosophila*

**DOI:** 10.1111/dgd.12604

**Published:** 2019-04-07

**Authors:** Shizue Ohsawa

**Affiliations:** ^1^ Laboratory of Genetics Graduate School of Biostudies Kyoto University Kyoto Japan

**Keywords:** cell–cell communication, *Drosophila*, oncogenic polarity‐deficient cells, tumor suppressive cell competition

## Abstract

Normal epithelial tissues often put anti‐tumorigenic pressure on newly emerged oncogenic cells through cell–cell communications. In *Drosophila* epithelium, clones of oncogenic cells mutant for evolutionarily conserved apico‐basal polarity genes such as *scribble* (*scrib*) and *discs large* (*dlg*) are actively eliminated when surrounded by normal cells. It has been reported that c‐Jun N‐terminal kinase (JNK) signaling in polarity‐deficient cells is crucial for their cell death. However, the mechanism by which normal epithelial tissues exert anti‐tumorigenic effects on polarity‐deficient cells had been elusive. Here, I describe our genetic studies in *Drosophila* epithelium especially focused on the role of surrounding normal epithelial cells in response to the emergence of polarity‐deficient cells. Furthermore, I also describe recent studies regarding the mechanism by which polarity‐deficient cells are extruded from the tissue, and discuss future perspectives on the study of cell–cell communications in epithelial homeostasis.

## Graphical abstract

Normal epithelial tissue eliminates newly emerged oncogenic polarity‐deficient cells through tumor suppressive cell competition. In this review, I summarize recent progress on the mechanism of this tumor suppressive cell competition in Drosophila, especially focused on the role of normal epithelial cells and describe future perspectives.

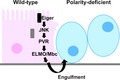

## 1 INTRODUCTION

Normal development and tissue homeostasis in multicellular organisms are robustly regulated through cell–cell interactions that coordinate cell proliferation and cell death. In particular, normal epithelial tissues often exert anti‐tumor effects against neighboring cells that acquire oncogenic alterations (Bissell & Radisky, 2001). For instance, oncogenic cells activating an oncogene such as Ras, Src, ErbB2, or YAP are eliminated from the epithelial layer when surrounded by wild‐type cells in mammals (Chiba et al., 2016; Hogan et al., 2009; Kajita et al., 2010; Leung & Brugge, 2012). Similarly, oncogenic cells mutant for an evolutionarily conserved apico‐basal polarity gene such as *scribble* (*scrib*) or *discs large* (*dlg*) are eliminated from the *Drosophila* imaginal epithelium when surrounded by wild‐type cells (Brumby & Richardson, 2003; Woods & Bryant, 1991). The removal of surrounding wild‐type cells by genetically inducing cell death allows these polarity‐deficient mutant cells to overgrow (Brumby & Richardson, 2003), suggesting that polarity‐deficient cells are actively eliminated from the epithelial tissue through “competitive” interactions with surrounding wild‐type cells, a form of “cell competition” (Claveria & Torres, 2016; Di Gregorio, Bowling, & Rodriguez, 2016; Madan, Gogna, & Moreno, 2018; Morata & Ripoll, 1975; Nagata & Igaki, 2018). Interestingly, MDCK cells downregulating *scrib* are also eliminated from the epithelial layer when surrounded by normal MDCK cells (Norman et al., 2012), suggesting that elimination of polarity‐deficient cells could be mediated through an evolutionarily conserved tumor suppressive cell competition. It has been shown that Eiger (the *Drosophila* tumor necrosis factor; TNF)‐JNK signaling drives cell death in polarity‐deficient cells in the *Drosophila* epithelium (Brumby & Richardson, 2003; Igaki, Pagliarini, & Xu, 2006; Igaki, Pastor‐Pareja, Aonuma, Miura, & Xu, 2009). In this review, I describe our findings especially in the role of surrounding normal epithelial cells in response to the emergence of polarity‐deficient cells in *Drosophila* imaginal epithelium. I also describe recent studies about the mechanism of cell extrusion that is an important process of tumor suppressive cell competition. I finally discuss future perspectives on the study of cell–cell communication in epithelial homeostasis.

## 2 EPITHELIAL CELLS PROMOTE ELIMINATION OF POLARITY‐DEFICIENT CELLS THROUGH JNK‐MEDIATED ENGULFMENT

**Figure 1:**
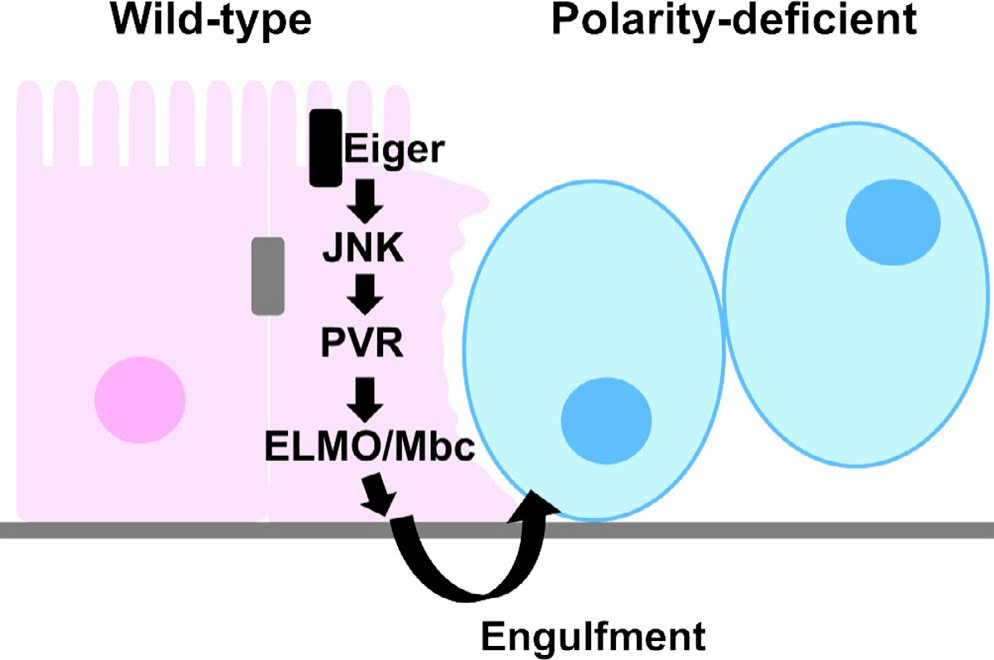
Epithelial cells promote elimination of polarity‐deficient cells through Jun N‐terminal kinase (JNK)‐mediated Engulfment. In response to the emergence of oncogenic polarity‐deficient cells, surrounding wild‐type cells activate JNK signaling, which activates PVR‐ELMO/Mbc engulfment pathway. See text for details

To understand the mechanism by which normal epithelial cells exert anti‐tumor effects against oncogenic polarity‐deficient cells, we analyzed the spatial pattern of cell elimination in the *Drosophila* eye‐antennal imaginal epithelium bearing *scrib* mutant cells (Ohsawa et al., 2011). Interestingly, most of dying *scrib* cells were restricted to the boundaries between *scrib* and wild‐type populations or incorporated into the wild‐type populations. In addition, live‐imaging analyses in the organ‐cultured eye‐antennal imaginal discs revealed that *scrib* cells were fragmented after they are detached from their clones and incorporated into wild‐type population, suggesting that *scrib* cells are killed by surrounding wild‐type cells through engulfment. Indeed, incorporated *scrib* cells within wild‐type populations were labeled by Lysotracker, a phagosome maturation marker, which means that *scrib* cells are engulfed by surrounding wild‐type cells. Genetic analyses revealed that this engulfment is triggered by JNK activation in surrounding normal cells in response to the emergence of polarity‐deficient cells. JNK signaling in surrounding normal cells induces the expression of the *Drosophila* PDGF/VEGF receptor PVR, which induces engulfment of oncogenic neighbors through the ELMO/Mbc engulfment pathway (Figure 1). Interestingly, live cell engulfment such as entosis, emperipolesis, and cannibalism is prevalent in human cancers (Durgan & Florey, 2018). The most common fate for entotic cells is cell death in mammary epithelial cancer cell lines (Overholtzer et al., 2007). It is possible that elimination of tumor cells through engulfment could be an evolutionarily conserved intrinsic tumor suppression mechanism in epithelial tissues.

## 3 THE LIGAND‐RECEPTOR SYSTEM SAS‐PTP10D DRIVES ELIMINATION OF POLARITY‐DEFICIENT CELLS

**Figure 2:**
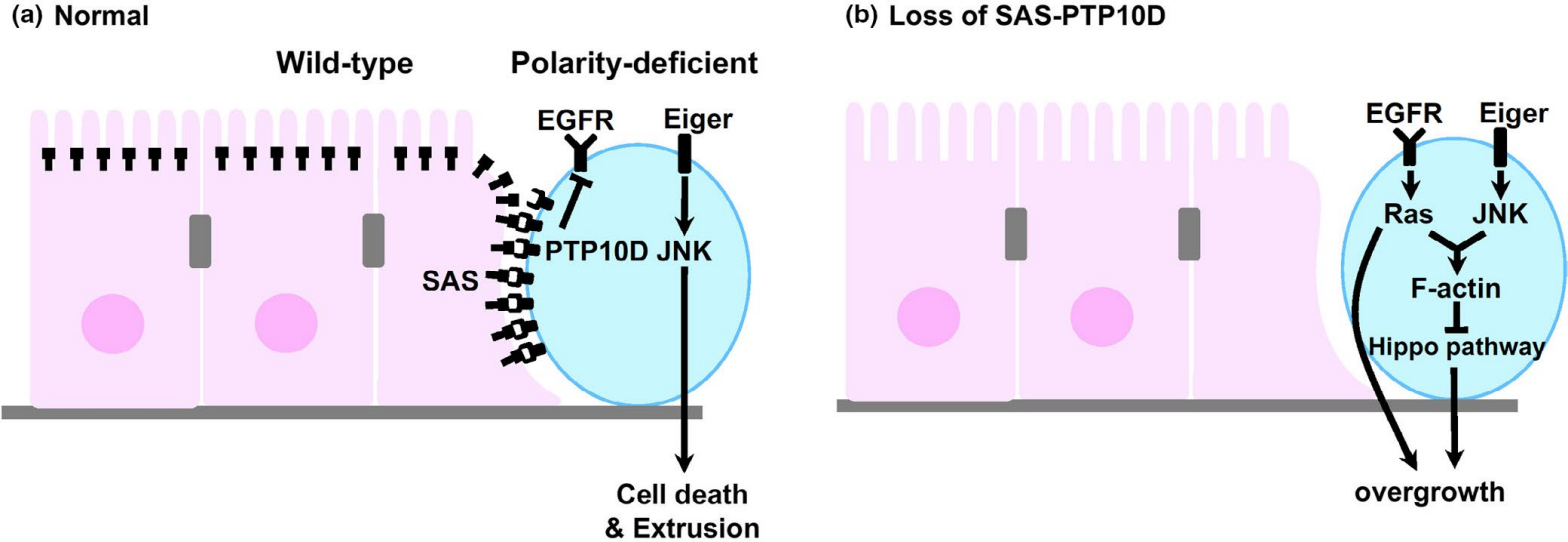
Sas‐PTP10D drives elimination of polarity‐deficient cells. (a) Jun N‐terminal kinase (JNK) signaling induces elimination of polarity‐deficient cells by SAS/PTP10D‐mediated inhibition of EGFR signaling. (b) In the absence of SAS/PTP10D system, EGFR signaling switches JNK signaling from cell death to overgrowth. See text for details

To reveal the initial mechanism by which normal epithelial tissues recognize oncogenic neighbors, we have conducted a genetic screen using a chemical mutagen EMS (ethyl methanesulfonate) for genes required for wild‐type cells to eliminate neighboring *scrib* cells. By genetic analyses and cDNA sequencings, we identified a cell surface ligand Sas that is required for normal epithelial cells to recognize polarity‐deficient cells (Yamamoto, Ohsawa, Kunimasa, & Igaki, 2017). Interestingly, while Sas is normally localized at the apical surface of epithelial cells, Sas in wild‐type epithelial cells surrounding polarity‐deficient cells relocalizes to the lateral cell surface at the interface with polarity‐deficient cells. This relocalization of Sas in surrounding cells to eliminate neighboring oncogenic cells prompted us to identify the Sas receptor expressed in polarity‐deficient cells. In addition to a transmembrane domain, Sas bears two extracellular domains, von Willebrand factor type C (VWC) and fibronectin type 3 (FN3) domains, which can homophilically interact with the same domain in other proteins. We performed RNAi (RNA interference) screen to identify the Sas receptor in polarity‐deficient cells, by targeting transmembrane proteins bearing either VWC or FN3 domain. This RNAi screen identified an evolutionarily conserved receptor‐type tyrosine phosphatase (RPTP), PTP10D (a PTPRJ homolog) as the Sas receptor that transmits the signal from normal epithelial cells. It has been reported that Sas and PTP10D interact and regulate longitudinal axon guidance in the *Drosophila* nervous system (Lee, Cording, Vielmetter, & Zinn, 2013). Strikingly, while PTP10D is normally localized at the apical surface of epithelial cells, PTP10D in polarity‐deficient cells relocalizes to the lateral cell surface at the interface with normal cells. Thus, when polarity‐deficient cells are generated in the epithelial tissue, Sas in normal cells and PTP10D in polarity‐deficient cells relocalize laterally at the interface between these cells, thereby driving elimination of polarity‐deficient cells through transactivation of Sas‐PTP10D signaling. Subsequent genetic analyses revealed that transactivation of Sas‐PTP10D signaling restrains EGFR (Epidermal Growth Factor Receptor) signaling in polarity‐deficient cells, therefore independently activating JNK signaling causes their cell death (Figure 2a). In the absence of Sas‐PTP10D signaling, EGFR‐Ras signaling is activated in polarity‐deficient cells. This upregulated EGFR‐Ras signaling cooperates with JNK signaling to accumulate F‐actin, thereby inactivating the tumor suppressor Hippo pathway, leading to an escape from cell elimination and overgrowth of polarity‐deficient cells (Figure 2b).

## 4 A SECRETED PROTEIN SERPIN5 IS REQUIRED FOR ELIMINATION OF POLARITY‐DEFICIENT CELLS

**Figure 3:**
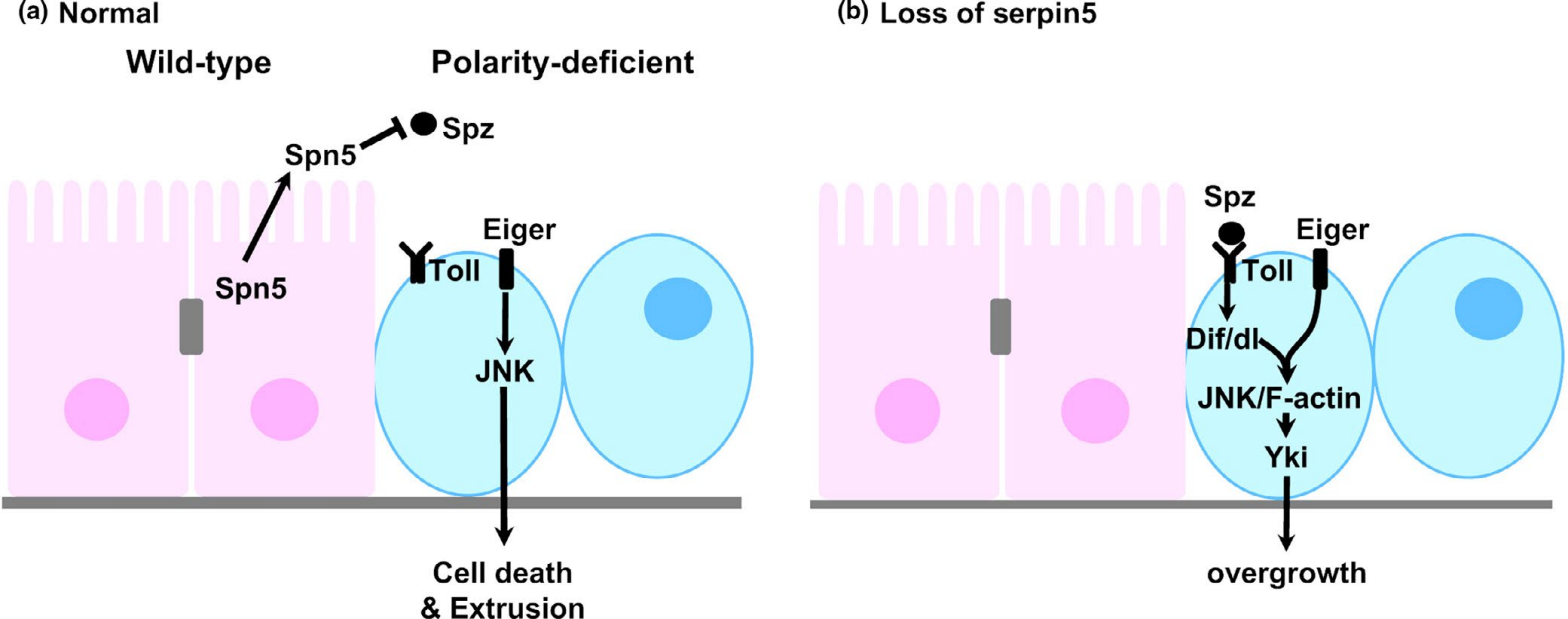
Serpin5 is required for elimination of polarity‐deficient cells. (a) Secreted Serpin5 from epithelial cells facilitates elimination of polarity‐deficient cells by inhibiting activation of Toll signaling in polarity‐deficient cells. (b) In the absence of Serpin5, extracellular Spz activates Toll signaling in polarity‐deficient cells, which results in their overgrowth. See text for details

In addition to Sas, the EMS‐based genetic screen also identified Serpin5 as a factor expressed in normal cells to facilitate elimination of polarity deficient cells (Katsukawa, Ohsawa, Zhang, Yan, & Igaki, 2018). Serpin5 is a secreted protein that negatively regulates Toll signaling by inhibiting proteolytic activation of the Toll ligand, Spaetzle (Spz) (Ahmad, Sweeney, Lee, Sweeney, & Gao, 2009). Importantly, when *Serpin5* is knocked down in *scrib* cells, elimination of *scrib* cells is also suppressed, as *Serpin5* is knocked down in surrounding wild‐type cells, suggesting that extracellular Serpin5 produced by normal and polarity‐deficient epithelial cells has an important role in elimination of polarity‐deficient cells (Katsukawa et al., 2018). Indeed, knockdown of *Serpin5* in both *scrib* cells and wild‐type cells causes stronger suppression of *scrib* cell elimination. The following genetic analyses revealed that extracellular Serpin5 antagonizes Toll signaling activation in polarity‐deficient cells (Figure 3a). Strikingly, forced activation of Toll signaling by overexpressing Toll, Dorsal (Dl), Dif or Persephone (Psh), or Toll‐related receptor (TRR) signaling by overexpressing Toll‐9 within *scrib* clones not only significantly suppressed *scrib* cell elimination, but also caused their tumorous growth caused by suppressing cell death and promoting cell proliferation. Mechanistically, elevated Toll signaling in *scrib* cells leads to JNK activation and F‐actin accumulation, which cause Yorkie (Yki) activation to suppress cell death and promote cell proliferation (Figure 3b). Thus, Serpin5 functions as an extracellular surveillance system by negatively regulating Toll signaling in polarity‐deficient cells.

## 5 JNK SIGNALING EXTRUDES POLARITY‐DEFICIENT CELLS THROUGH AUTOCRINE SLIT‐ROBO2 REPULSIVE SYSTEM

**Figure 4:**
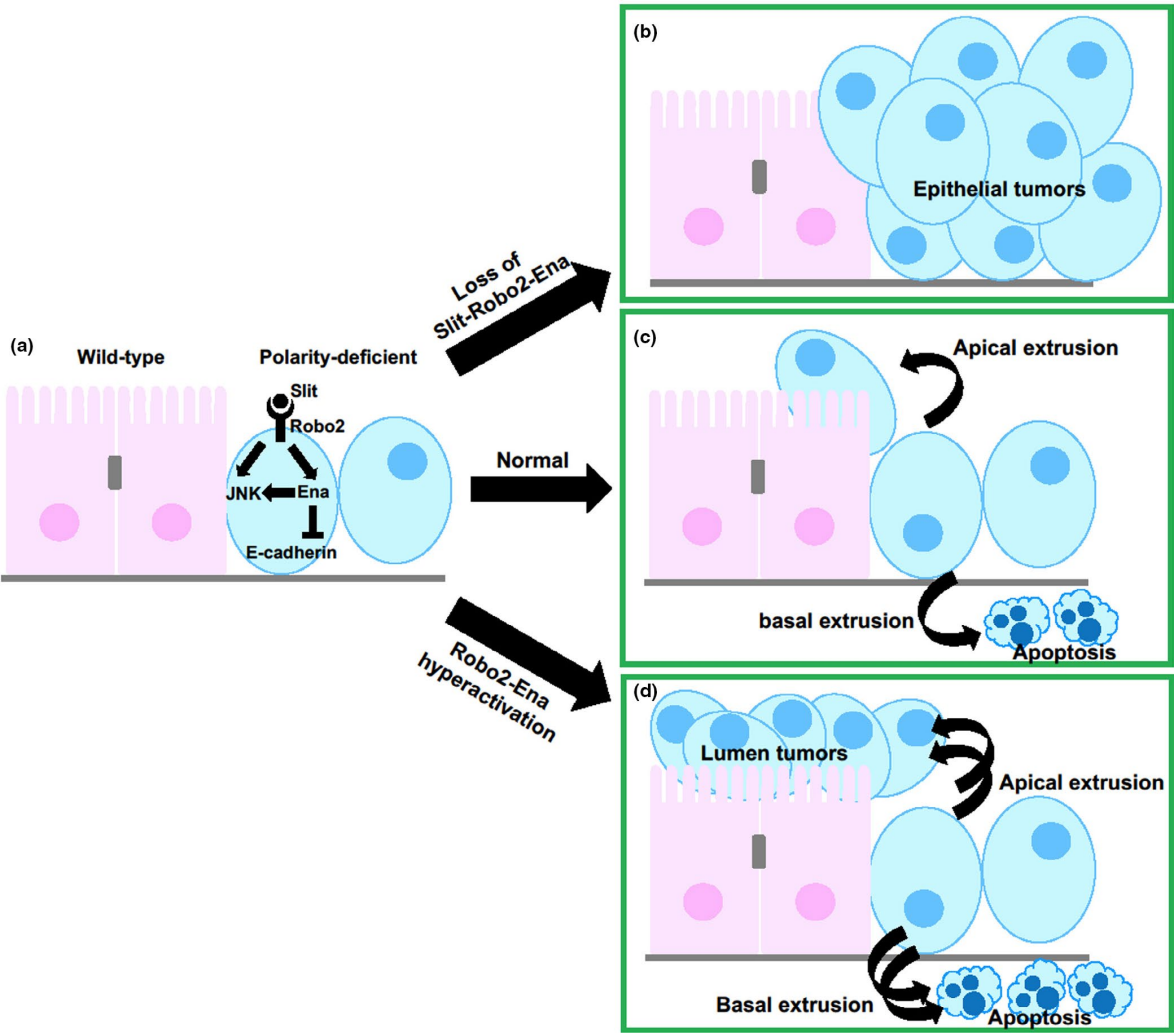
Jun N‐terminal kinase (JNK) signaling extrudes polarity‐deficient cells through autocrine Slit‐Robo2 repulsive system. In polarity‐deficient cells surrounded by wild‐type cells, JNK activates Slit‐Robo2‐Ena signaling leading to downregulation of E‐cadherin (a). In this normal situation, polarity‐deficient cells are predominantly basally extruded and cause apoptotic cell death (c). In the absence of Slit‐Robo2‐Ena signaling, polarity‐deficient cells escape from extrusion, leading to their overgrowth in the epithelial layers (b). Conversely, hyperactivation of Robo2‐Ena signaling induces excess extrusion and luminal tumors (d). See text for details

As mentioned above, JNK signaling in polarity‐deficient cells is crucial for their cell death in the presence of wild‐type cells. Interestingly, it has been recently reported that prior to inducing cell death, JNK signaling drives extrusion of polarity‐deficient cells from the epithelial layer, (Vaughen & Igaki, 2016). A dominant modifier screen and subsequent genetic analyses revealed that an evolutionarily conserved repulsive axon guidance signaling, the secreted ligand Slit‐Robo system and its downstream cytoskeletal effector Enabled (Hogan et al., 2009)/VASP (Araujo & Tear, 2003; Brose & Tessier‐Lavigne, 2000), acts downstream of JNK to induce extrusion of polarity‐deficient cells by disrupting E‐cadherin. Importantly, Robo2‐Ena signaling also activates JNK signaling, indicating that JNK and Robo2‐Ena modules form positive feedback loop to amplify extrusive signaling (Figure 4a). By this JNK and Slit‐Robo2‐Ena signaling, basally extruded *scrib* cells are rapidly removed by apoptosis, while apically extruded *scrib* cells into the lumen are not universally dying (Figure 4c). Inactivation of Slit‐Robo2‐Ena signaling suppresses extrusion of *scrib* cells, which results in their overgrowth in the epithelial layer (Figure 4b). Interestingly, hyperactivation of Robo2‐Ena signaling causes excess extrusion and the following luminal tumorigenesis of *scrib* cells (Figure 4c). Thus, Slit‐Robo2 system exerts both tumor suppressive and tumor promotive effects by regulating extrusion of oncogenic polarity‐deficient cells.

## 6 PERSPECTIVES

Our recent genetic studies reveal the mechanism of tumor suppressive cell competition between oncogenic polarity‐deficient cells and wild‐type cells. However, various questions remain elusive. For instance, it is unknown how Sas and PTP10D relocalize to the lateral cell surface of epithelial cells at the clone interface between normal and polarity‐deficient cells. We found that apical proteins such as Bazooka, aPKC, and Patj and sub‐apical protein E‐cadherin also relocalize to the lateral surface at the clone interface, as well as Sas and PTP10D (Yamamoto et al., 2017). These observations suggest that epithelial cells might expand their apical surface to the lateral region at the clone interface, which enables Sas and PTP10D to interact with each other *in trans* at the clone interface.

It is also unknown why polarity‐deficient cells are more sensitive to “loss of Serpin5” to elevate Toll signaling than surrounding wild‐type cells. It has been reported that JNK signaling activates Toll signaling in *Drosophila* imaginal epithelia (Wu et al., 2015). Thus, JNK signaling might induce Toll activation in polarity‐deficient cells in response to “loss of Serpin5”.

Furthermore, the other question is how the fate of polarity‐deficient cells is determined after extrusion from epithelial tissue by cell competition: Why do basally extruded polarity‐deficient cells undergo apoptosis (Nakajima, Meyer, Kroesen, McKinney, & Gibson, 2013; Vaughen & Igaki, 2016), while apically extruded polarity‐deficient cells can escape apoptosis and exhibit overgrowth phenotype by hyperactivating Robo2‐Ena signaling (Vaughen & Igaki, 2016)? Similarly, it has been reported in the *Drosophila* wing imaginal disc that *scrib‐RNAi* cells are basally extruded, followed by apoptosis in the “tumor coldspots” region, while *scrib‐RNAi* cells are apically extruded and cause tumorigenic overgrowth through JAK/STAT signaling in the “tumor hotspots” region (Tamori, Suzuki, & Deng, 2016). A plausible reason is that circulating blood cells, termed hemocytes, are recruited to the basal side of epithelial sheets (Pastor‐Pareja, Wu, & Xu, 2008). Interestingly, it has been shown that hemocytes produce and secrete Eiger, which stimulates cell death in polarity‐deficient cells (Parisi, Stefanatos, Strathdee, Yu, & Vidal, 2014). In addition, hemocytes have been shown to secrete Eiger and amplify JNK signaling in Ras‐activating polarity‐deficient tumors, leading to their tumorous overgrowth (Cordero et al., 2010; Perez, Lindblad, & Bergmann, 2017). Further studies would provide novel insights in epithelial homeostasis through cell–cell communications.
